# Identifying the African Wintering Grounds of Hybrid Flycatchers Using a Multi–Isotope (*δ* ^2^H, *δ* ^13^C, *δ* ^15^N) Assignment Approach

**DOI:** 10.1371/journal.pone.0098075

**Published:** 2014-05-21

**Authors:** Thor Veen, Mårten B. Hjernquist, Steven L. Van Wilgenburg, Keith A. Hobson, Eelke Folmer, Laura Font, Marcel Klaassen

**Affiliations:** 1 Biodiversity Research Centre, University of British Columbia, Vancouver, British Columbia, Canada; 2 Theoretical Biology Group, University of Groningen, Groningen, Groningen, The Netherlands; 3 Animal Ecology, Uppsala University, Uppsala, Uppland, Sweden; 4 Environment Canada, Saskatoon, Saskatchewan, Canada; 5 Department of Marine Ecology, Royal Netherlands Institute for Sea Research, Den Burg, Noord-Holland, The Netherlands; 6 Deep Earth Cluster, Vrije Universiteit Amsterdam, Amsterdam, Noord-Holland, The Netherlands; 7 Department of Animal Ecology, Netherlands Institute of Ecology, Wageningen, Gelderland, The Netherlands; 8 Centre for Integrative Ecology, Deakin University, Geelong, Victoria, Australia; Scottish Association for Marine Science, United Kingdom

## Abstract

Migratory routes and wintering grounds can have important fitness consequences, which can lead to divergent selection on populations or taxa differing in their migratory itinerary. Collared (*Ficedula albicollis*) and pied (*F. hypoleuca*) flycatchers breeding in Europe and wintering in different sub-Saharan regions have distinct migratory routes on the eastern and western sides of the Sahara desert, respectively. In an earlier paper, we showed that hybrids of the two species did not incur reduced winter survival, which would be expected if their migration strategy had been a mix of the parent species' strategies potentially resulting in an intermediate route crossing the Sahara desert to different wintering grounds. Previously, we compared isotope ratios and found no significant difference in stable-nitrogen isotope ratios (*δ* ^15^N) in winter-grown feathers between the parental species and hybrids, but stable-carbon isotope ratios (*δ* ^13^C) in hybrids significantly clustered only with those of pied flycatchers. We followed up on these findings and additionally analyzed the same feathers for stable-hydrogen isotope ratios (*δ* ^2^H) and conducted spatially explicit multi-isotope assignment analyses. The assignment results overlapped with presumed wintering ranges of the two species, highlighting the efficacy of the method. In contrast to earlier findings, hybrids clustered with both parental species, though most strongly with pied flycatcher.

## Introduction

The link between breeding, stopover and wintering sites can have important fitness consequences for migrating individuals. The potential fitness consequence of migration are many-fold and involves the interaction of direction, distance and duration of migration, physiological state, and use of stop-over sites and wintering grounds that may vary in quality [1]–[5]. The same factors may lead to macro-evolutionary responses if populations or taxa differ in their migratory trajectory and experience different selective forces during migration and on the wintering grounds [6].

Migratory divides are an example of such segregation and refers to the situation where there are distinctly different migratory routes and wintering locations segregating populations or taxa. These divides occur in a variety of taxa, e.g. the northern flickers [7], Swainson's thrush [8], Eurasian reed warbler [9] and greenish warbler [10] and we use one of the best-studied species, the blackcap (*Sylvia atricapilla*), to exemplify how migratory divides can result in divergent selection among populations. Two blackcap populations breeding in central Europe differ in their migratory routes; individuals of one follow a southeastern route to east Africa and of the other a western route to wintering grounds in West Africa. The migratory direction is genetically determined [11] and can change, as a novel migratory strategy evolved towards north-western wintering grounds in Britain over the last five decades [12], [13]. Gene flow between populations differing in migratory strategy was reduced as hybrids exhibited an intermediate migration direction, which is hypothesised to be maladaptive [11], [12]. Furthermore, individuals from different wintering populations differ in their arrival time at the breeding grounds in central Europe, leading to wintering-ground based assortative mating and habitat choice [6], [14], [15], also resulting in genetic and phenotypic divergence [16].

A key component of understanding the evolutionary consequences of migratory divides, and migratory routes and behaviours in general [17], is estimating the fitness consequences of the different migratory strategies. Comparison of the fitness of hybrids with intermediate migratory behaviour with the fitness of the parental species could help to develop understanding of the evolution of migration. However, fitness measures of blackcap hybrids are lacking [15], [18]. Strong fitness effects of migratory divides are reported from other systems. For example, hybrids between willow warbler subspecies (*Phylloscopus trochilus trochilus* and *P. t. acredula*), which have distinct migratory routes along the east and west coast of Africa, are thought to follow an intermediate route through the Sahara and perish [19]. Although strong selection against hybrids is evident, the causal mechanisms are less clear. Selection against hybrids may result from the use of sub-optimal intermediate migratory routes, but other factors differing between the wintering grounds (e.g. food availability, predation, pathogens) or intrinsic low viability (post fledging) of hybrids cannot be excluded.

The study of species with divergent migratory routes and their hybrids in combination with fitness data provide opportunity to develop insight into the determinants of migratory behaviour. Hybridizing collared (*Ficedula albicollis*) and pied (*F. hypoleuca*) flycatchers provide such an opportunity. Despite separate migratory routes (eastern versus western Africa, respectively) and wintering grounds (southern and western Africa, respectively) of the parental species, hybrids have not been found to incur reduced winter survival [20], [21] (and references therein). In an earlier study [21] we used stable isotope ratios of carbon (*δ* ^13^C) and nitrogen (*δ* ^15^N) in feathers to investigate whether differences in wintering grounds of both parental species were reflected in their isotope values. Subsequently, we used the feather isotope values of the hybrids to help infer their wintering grounds and most likely migration route. We found a clear difference in *δ* ^13^C values of feathers grown on the wintering grounds between the two species, but no difference in *δ* ^15^N values. Stable-carbon isotope ratios in feathers of hybrids were similar to those of pied flycatchers and differed significantly from those of collared flycatchers. Stable- nitrogen isotope values of hybrids were similar to both parental species. Based on these results, we suggested that hybrids utilise the same winter sites as pied flycatchers and hypothesised that the predicted maladaptive consequences of an intermediate route might be circumvented by hybrids following the pied flycatcher's western migratory route. However, differences in feather *δ* ^13^C values do not necessarily reflect spatial differences in wintering ground locations as this isotope also reflects diet and microhabitat [5], [22].

Hydrogen stable isotope ratios (*δ* ^2^H) are increasingly used for geographical assignments as their isoscapes show relatively continuous patterns on large, continental scales (see [23] and references therein). This benefit of *δ* ^2^H combined with the finding that distinct multi-isotope (*δ* ^13^C, *δ* ^15^N, *δ* ^2^H) regions of Africa could be used to infer locations of moult on the wintering grounds [24] suggests that our previous analyses could be expanded upon by incorporating an additional intrinsic marker into assignments to wintering ground moult origins. Our primary aim for adding *δ* ^2^H measurements was to investigate the robustness of our earlier conclusion that wintering ranges of pied and hybrid flycatchers overlap. Validating this conclusion is important as it provides the main support for our hypothesis that the lack of expected lower overwinter survival of hybrids may be the consequence of sharing the same wintering location and potentially also a migratory trajectory of one of the parental species. We did this by applying spatially explicit multi-isotope assignment of individuals to geospatial models of the expected isotopic composition of feathers grown on the wintering regions (sub-Saharan Africa), based upon analysis of *δ* ^2^H, *δ* ^13^C, and *δ* ^15^N values in feathers. This approach allowed us to better assess the spatial overlap in the presumed wintering grounds of the collared and pied flycatcher and determine potential wintering regions for the hybrid flycatchers in relation to those of the parental species.

## Material and Methods

### Feather samples

The median tertial feather was collected for stable isotope analyses from collared, pied and hybrid flycatchers on the island of Gotland (Sweden) in the Baltic Sea at the end of the breeding season in 2004 and 2005. This feather is moulted on the wintering grounds [20], [25] and isotope values therefore represent local environmental conditions at these sites. On the basis of a combination of morphological and behavioural measurements, validated using molecular techniques [21], [26], each individual was assigned to be a pure collared flycatcher (n = 36), pure pied flycatcher (n = 24) or a hybrid (n = 15). The hybrid sample consisted of a mixture of individuals from pairings between collared flycatcher females and pied flycatcher males (n = 5) and pied flycatcher females and collared flycatcher males (n = 10). These were the same individuals as used in Veen *et al.* [21], with the exclusion of two collared flycatchers for which the analysis of *δ* ^2^H_f_ failed. For one collared flycatcher and one hybrid a feather sample from 2005 instead of 2004 was used.

### Stable isotope analyses

Values of *δ* ^13^C and *δ* ^15^N in feather samples (washed in chloroform) and casein standards were analyzed at the Netherlands Institute of Ecology in 2006 [21]. Measurements were determined using a HEKAtech EuroEA elemental analyzer coupled via a Finnigan con-flo interface to a Finnigan Delta S isotope ratio mass spectrometer. Measurements were reported relative to the international standards AIR for *δ* ^15^N and Vienna PeeDee Belemnite (VPDB) for *δ* ^13^C. Replicate measurements of the standards were within 0.2 ‰ for both *δ* ^13^C and *δ* ^15^N.

Two years after the *δ* ^13^C and *δ* ^15^N analyses, in 2008, feather δ^2^H measurements were conducted on a Thermo Finnigan Delta XP mass spectrometer equipped with a TC-EA pyrolysis furnace in the Faculty of Earth and Life Sciences at the Vrije Universiteit Amsterdam, The Netherlands. Parts of the same feathers as used in Veen *et al.* [21] were used for this analysis. Feather samples and keratin standards (∼0.1–0.15 mg, see below) were placed into silver capsules and introduced into the TC-EA reactor at 1450°C resulting in quantitative conversion to H_2_ gas, which was separated from other pyrolysis products in a GC column and subsequently analyzed in the mass spectrometer. A total of 18 keratin standards and 27 samples were analyzed in each run. Each feather sample was analyzed in triplicate, thus nine feather samples were analysed per run. The reported hydrogen isotope values of the feather samples are the mean value of the triplicate analysis. In addition, internationally reference standard IAEA CH7 was analyzed (n = 4 per run) to control instrument performance. Hydrogen isotope ratios of the feathers are reported for non-exchangeable H relative to the V-SMOW scale (‰) and were calibrated using the ‘comparative equilibration approach' with pre-calibrated keratin standards CFS (−147±5 ‰), CHS (−187±2 ‰) and BWB-II (−108±4 ‰) as described in Wassenaar & Hobson [27]. Replicate measurements of these standards were within 4 ‰.

### Geographic assignments to moult origin

We assigned the individual flycatchers to their wintering ground moult origins on the basis of the isotopic composition (*δ* ^2^H, *δ* ^13^C, and *δ* ^15^N) of their feathers. To this end, we used the feather isotope data to conduct spatially explicit assignments to maps (hereafter ‘isoscapes’) of the predicted isotopic composition (*δ* ^2^H, *δ* ^13^C, and *δ* ^15^N) of feathers, as shown by Hobson *et al.* [24]. Feather isoscapes were derived from isoscapes reflecting precipitation amount-weighted mean growing-season *δ* ^2^H in precipitation (*δ* ^2^H_p_, [28]), the theoretical spatial *δ* ^13^C distribution of plants in Africa ([29], and a plant *δ* ^15^N isoscape developed by Craine *et al.* [30]. Calibration of these separate isoscapes into a cluster analysis describing four discrete multi-isotope feather isotope zones in Africa was first reported in Hobson *et al.* [24]. Hobson *et al.* [24] used regression parameters derived from regression of *δ* ^2^H in Reed Warbler feathers (*δ* ^2^H_f_) against *δ* ^2^H_p_ to convert the *δ* ^2^H_p_ isoscape of Bowen *et al.* [28] into a *δ* ^2^H_f_ isoscape for Africa based upon data reported in Procházka *et al.* [9]. We used the feather *δ* ^13^C and *δ* ^15^N isoscapes calibrated by Hobson *et al.* [24] from plant *δ* ^13^C and *δ* ^15^N isoscapes using a discrimination of +2 *‰* to the *δ* ^13^C isoscape to account for plant-feather discrimination and a discrimination of +5 *‰* between plant and feathers was applied to the plant *δ* ^15^N isoscape of Craine *et al.* [30] (for details see Hobson *et al.* [24]). Following Hobson *et al.* [31], Wunder [32] and Van Wilgenburg and Hobson [33], each individual flycatcher was assigned to its most likely moult origin on the basis of a multivariate normal probability density function (hereafter mvnpdf) as first described by Royle and Rubenstein [34].

We used the mvnpdf to assess the likelihood that a given geo-referenced location (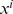) within the feather isoscapes (resolution of 0.33°) for sub-Saharan Africa (excluding Madagascar) represented a potential origin as follows:
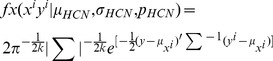



Here, 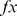 represents the spatially explicit probability density function for 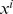 representing the location of origin given the feather of unknown origin (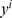) with a measured isotopic composition (*δ* ^2^H, *δ* ^13^C, *δ* ^15^N). The expected mean, standard deviation, and pairwise correlations of *δ* ^2^H, *δ* ^13^C, *δ* ^15^N are denoted as 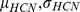 and 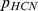 respectively, for a feather grown at that indexed location (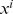). The parameter 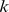 represents the number of isotopes. For each location, estimated mean isotopic composition was estimated directly from raster cells in the calibrated isoscapes for *δ* ^2^H, *δ* ^13^C, and *δ* ^15^N, and thus 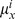 represents a vector of means for each geographic location (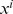) being considered:
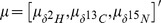



Finally, the term 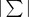 represents the variance-covariance matrix:
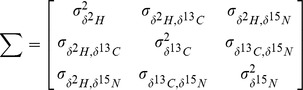



Variance for a given isotope is represented in the diagonal elements of the matrix, while off-diagonal elements represent covariance between the denoted isotope pairs. Following Royle and Rubenstein [34] and Flockhart *et al.* [35], we assumed spatial stationarity of covariance across the isoscapes; that is, we assumed that the variance-covariance between isotopes did not change across the wintering range.

This assumption may become a problem if the covariances between the isotopes vary between subpopulation (e.g. due to human activities such as agriculture), but the data and statistical methodologies to allow non-stationary variance-covariance matrices are currently lacking.

Geographic locations that fell within the upper 67% of the spatially explicit probability densities within the likelihood map (based upon mvnpdf assessment) for a given individual were selected and coded as 1; all others were coded as 0 [31], [32]. Thus, for each individual being assigned to origin, we obtained one binary map. Finally, we depicted the likely population origin by summing over all individual binary surfaces for each species separately. To aid interpretation, the resulting assignment maps were rescaled by division by the maximum number of individuals assigned to it a single cell (pixel), to obtain maps depicting the relative proportion of samples that were isotopically consistent with a given cell within the isoscapes. The maximum number of individuals relative to the sample size was high for each group and analysis (mean proportion of 0.85 and range of 0.71–1). All analyses were conducted in the R (v 2.15.1) statistical computing environment [36]. Variance-covariance was estimated from the multi-isotope data for our unknown origin samples using the mvnmle package Gross and Bates [37], and spatially explicit assignments employed functions available in the raster package of Hijmans and Van Etten [38]. Our assignment algorithm does not unequivocally assign individuals to a single location, but instead assigns individuals to all pixels in the map that are statistically consistent (at the selected odds ratio) with the observed multi-isotopic composition of the feather, regardless of whether those pixels are adjacent or spatially distant. Therefore, a single individual can theoretically be simultaneously assigned to both wintering ranges or multiple countries within Africa. For example, an individual could be simultaneously assigned to pixels in Lesotho, southern Democratic Republic of Congo and Cote d'Ivoire based upon isotopic similarity with predicted values from the isoscapes. We were interested in assessing not only the geographic distribution of origins, but also the relative proportion of the hybrid sample that was potentially consistent with the parental species wintering range. Therefore, we used the minimum and the maximum numbers of individuals assigned to a given wintering range and divided these values by the hybrid sample size, thus yielding the minimum and maximum proportion of our sample that was isotopically consistent with a given wintering range given the assumptions of our assignment model.

### Ethics statement and data availability

The Swedish National Board for Laboratory Animals approved the collection of feathers (permit number M 78–05). The isotope data and isoscape maps used for the analyses in this paper and Veen *et al.* 2007 [21] are available from the Dryad Digital Repository: http://doi:10.5061/dryad.615tj.

## Results

**Figure 1:**
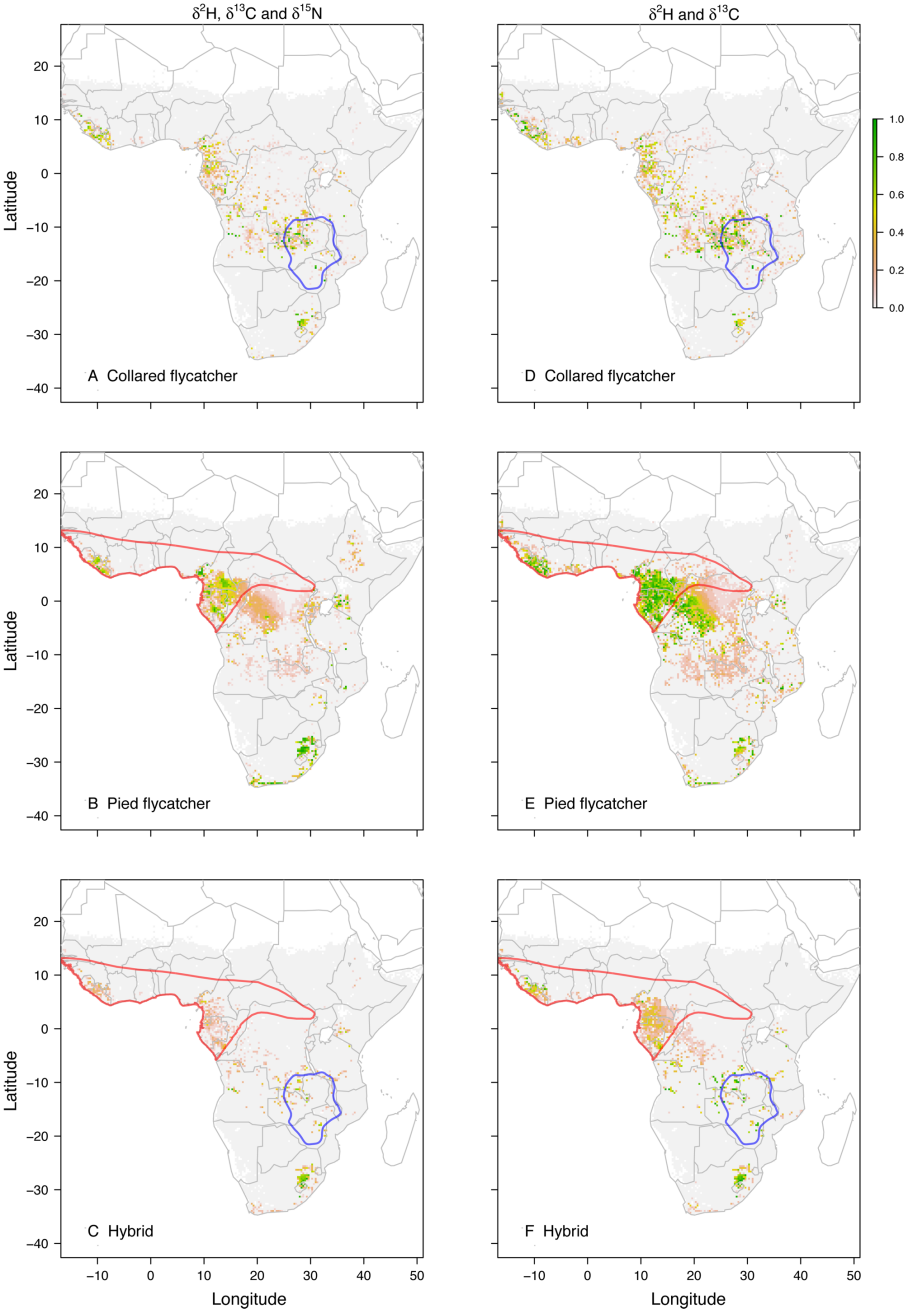
Predicted overwintering regions for collared, pied and hybrid flycatchers based on isotopes assignment. Predicted overwintering regions for collared (A), pied (B) and hybrid flycatchers (C) based on three isotopes (*δ* ^2^H, *δ* ^13^C and *δ* ^15^N) combined. The presumed overwinter grounds are shown in blue for the collared and red for the pied flycatcher (from BirdLife International, NatureServe [52]). Panel E-F show the predicted wintering ranges based on two isotopes (*δ* ^2^H and *δ* ^13^C). For each pixel (0.33°), the probability that the isotopes extracted from a feather originated from a given area is calculated and stored as a 1 if it falls within the upper 67% of the spatially explicit probability density maps of an individual (and as a 0 if not). The colours represent the proportion of individuals that got a 1 assigned to the cell and hence represent an indication of how likely each geographical location is as an overwintering site.

The regions showing the strongest match between the isoscapes and the δ^2^H, δ^13^C and *δ* ^15^N values of feathers (green regions on the multi-isotope assignment maps) showed some overlap with the presumed wintering grounds of the collared (Figure 1A) and considerable overlap for pied flycatchers (Figure 1B). Compared to single isotope assignments, one expects multi-isotopic assignments to put more conservative limits to distribution ranges only. Thus, as anticipated, regions with high support on the reference maps also occurred outside the presumed wintering ranges of collared and pied flycatcher, reflecting regions within the isoscapes that were isotopically similar to those in the presumed wintering ranges. The cluster of high assignment probability in South Africa is most likely driven by the high altitude of the particular region, linked with depleted precipitation.

The majority of collared flycatcher samples were associated with areas of the northwestern portion of their presumed wintering range (northwestern Zambia and the southern tip of the Democratic Republic of Congo). Analysis of the three isotopes in pied flycatcher feathers suggested there was a high likelihood that individuals wintered in southeastern portions of their presumed wintering range (Cameroon, Equatorial Guinea, Gabon and northern Congo). However, high likelihoods were also attributed to a small area in the western portions of their presumed wintering range (mainly Liberia; Figure 1B).

**Figure 2:**
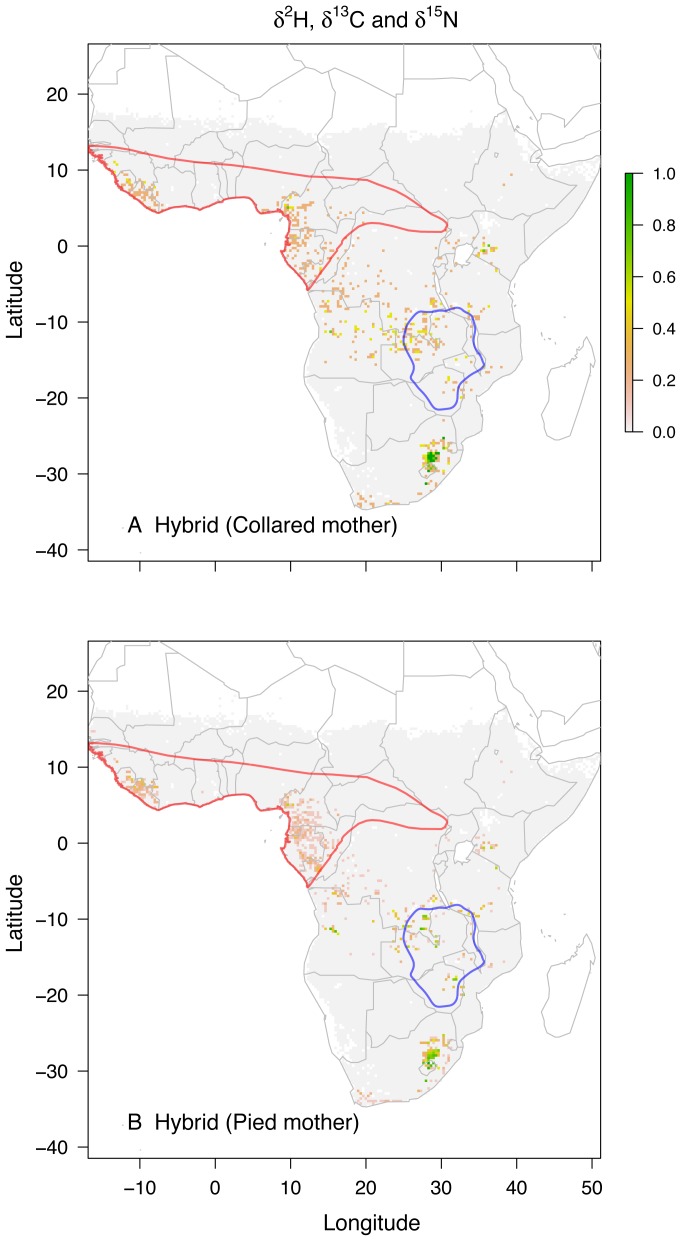
Predicted overwintering regions for the two types of flycatcher hybrids. Isotope assignments for hybrids with a collared (A) and pied flycatcher mother (B) based on three isotopes (*δ* ^2^H, *δ* ^13^C and *δ* ^15^N) combined. The presumed overwintering grounds of the two parental species are shown in blue for the collared and red for the pied flycatcher. For methodological details see legend of Figure 1 and main text.

Spatially explicit assignment to origins for hybrids (Figure 1C) showed the greatest consistency with the wintering grounds of the pied flycatcher. Approximately 30–70% of the assigned origins for hybrids were consistent with the expected pied flycatcher wintering range including southern west Africa, Cameroon, Equatorial Guinea, Gabon and northern Congo. In contrast, overlap of assigned origins for hybrids with the presumed collared flycatcher winter region was small and patchy, but locally with some cells being isotopically consistent with 100% of the samples. There was little difference between the assigned origins of maternal collared and pied flycatcher hybrids when depicted separately (Figure 2).

## Discussion

Our multi-isotope assignments of feathers from individual pied flycatchers were consistent with their presumed wintering grounds. A similar but less strong overlap was found for the collared flycatchers. For hybrids, the bight of Benin received good support as the origin of winter-grown hybrid feathers. This falls within the presumed wintering ranges of the pied flycatcher and (weakly) supports our earlier work [21]. However, the highest likelihood of origin was found in the presumed collared flycatcher wintering range, but only at few and discontinuous locations.

These two main wintering regions can be reached in two ways, through following one of the parental routes, or by following a novel intermediate route. The mechanisms underlying navigation during long-distance migration are still poorly understood [39], but given the often strong genetic determination of migratory behaviour [40]–[43], we will discuss the above two scenarios using different genetic processes.

Hybrids can reach the parental wintering grounds following an established migration route if the genetic underpinning of the route is not disrupted by recombination. Dominance and sex-linkage are two established mechanisms to achieve this. In the case of genetic dominance, the genetics underlying migratory behaviour of one species are dominant over the other, resulting in all hybrids migrating to the wintering location of the dominant parental species. This idea was put forward as an explanation for our earlier work [21] where isotopic values of hybrids exclusively clustered with those of pied flycatcher. If migratory behaviour was sex-linked and maternally inherited, hybrids would migrate to the same wintering ground as their mother. Unlike the former scenario, in this case hybrids would be found on the wintering grounds of both parental species. However, we found no support for this hypothesis in our earlier work (i.e. no statistically significant effect of maternal species of hybrids on isotope value [21].

If the genetic basis of the hybrids' orientation was inherited additively from the two parental species, we might expect an intermediate migration strategy. If these hybrids would orient using a ‘clock-and-compass’ mechanism [43], [44] it would presumably take them anywhere between Nigeria (within the pied flycatcher range) through potentially novel wintering grounds (Democratic Republic of Congo) east to Zambia (within the collared flycatcher range). This novel wintering grounds scenario is supported by regions of elevated likelihood of origin outside of the parental species ranges. However, it should be recognized that large areas of Africa are predicted to be isotopically similar [24], and hence elevated likelihood indicates merely a potentially suited wintering region. Yet, it provides important knowledge to guide future efforts to locate flycatchers on their wintering grounds.

As we did not find clear patterns of elevated likelihood of origin for hybrids, we unfortunately cannot draw firm conclusions where hybrids winter, what migratory route they follow, and how their migratory strategy is inherited.

Assignments of individuals and populations to isoscapes must also be interpreted cautiously owing to the potential errors and assumptions implicit in the isoscapes themselves. It is important to remember that the isoscapes are not data in and of themselves but represent static models of the expected geographic distribution of the isototope in question [45]. For example, the *δ* ^2^H isoscape for Africa created by Bowen *et al.* [28] is based upon data from only 44 stations of varying duration over the almost 50 year period of sampling by the Global Network for Isotopes in Precipitation (GNIP). In addition, other processes such as human alteration of natural habitats can alter the isotopic baselines of large-scale landscapes, which may or may not be accurately reflected in the models; for example, *δ* ^15^N is strongly influenced by anthropogenic factors such as agriculture [29], [30]. We tested for a potential bias this may introduce by excluding *δ* ^15^N from the analyses but found very similar results (Figure 1D-F).

Also, the assignment hinges on the assumption that the local isotopic composition is reflected in the locally grown feathers. Several studies have been validating this assumption in recent years [24], [46], [47] yielding variable results depending on species and isotopes used. Ideally, flycatcher winter-grown feather isotope values should be determined across their wintering ranges to validate our assignments and function as a reference map or calibration for assigning individuals caught on the breeding grounds to a more specific wintering location. This is not a trivial task, given the large presumed wintering grounds and difficulty of locating the birds (as suggested by the low number of ring recoveries) but efforts for the pied flycatcher in western Africa are ongoing (Christiaan Both, personal communication).

In parallel to improvements to the isoscape estimates, several other methods could improve our understanding of migratory behaviour of flycatchers. First, isotopic data can be combined with models of migratory direction derived from ring recoveries [33] to improve the assignments. For the flycatcher species this would have to focus on ring recoveries in Europe, as recoveries from the wintering grounds are very rare.

Secondly, individual tracking devices such as light-level geolocators that can even be carried by small birds the size of flycatchers, can be combined with isotope analyses to try to cross-validate the results of the isotope assignments (or *vice versa*) or improve the assignment to wintering grounds [47]–[49], but see [50] for a critical meta-analysis showing a negative effect of geolocators on fitness for small aerial insectivores). Geolocators furthermore provide valuable information on the migration route of the individual [8], which would be very informative for studies like ours, assuming migration route was not impacted by the presence of a geolocator. Combinations of different methods will become increasingly more accessible with the fast technological development of tracking devices and the decrease of costs of molecular work creating an exciting future for the study of migratory behaviour [51].

In summary, our study provides reasonable confirmation of the presumed wintering locations of pied and collared flycatchers and uncovered areas with similar isotopic composition, which could conceivably represent suitable (unknown) wintering grounds. Our assignments of hybrids to putative wintering ground origins suggested the strongest overlap with that of the pied flycatchers, but it is hard to draw firm conclusions. Future research utilizing individual tracking methods will hopefully yield further insights into the migratory routes of hybrids and whether they have their own unique wintering grounds, or whether they share their wintering grounds with one or both of the parental species.
